# A novel dual probe-based method for mutation detection using isothermal amplification

**DOI:** 10.1371/journal.pone.0309541

**Published:** 2024-10-22

**Authors:** Nidhi Nandu, Michael Miller, Yanhong Tong, Zhi-xiang Lu

**Affiliations:** Revvity, Inc., Waltham, MA, United States of America; Rutgers Biomedical and Health Sciences, UNITED STATES OF AMERICA

## Abstract

Cost efficient and rapid detection tools to detect mutations especially those linked to drug-resistance are important to address concerns of the rising multi-drug resistance infections. Here we integrated dual probes, namely a calibrator probe and an indicator probe, into isothermal amplification detection system. These two probes are designed to bind distinct regions on the same amplicon to determine the presence or absence of mutation. The calibrator probe signal is used as an internal signal calibrator for indicator probe which detects the presence or absence of the mutation. As an illustrative example, we evaluated the applicability of this dual probe method for detecting mutations associated with rifampicin (RIF) drug resistance at codons 516, 526 and 531 of the *rpo*B gene in *Mycobacterium tuberculosis*. In this assessment, we examined 127 artificial samples comprising wild types and mutants with single or multiple mutations. Our results demonstrated 100% accuracy for both wild types and mutants for mutations at codons 526 and 531. As regards to mutations at codon 516, the wild type was identified with 100% accuracy, while the mutants were identified with 95% accuracy. Moreover, when we extended our evaluation to include clinical MTB strains and the Zeptometrix MTB Verification panel, our method achieved 100% accuracy (5 out of 5) in identifying wild-type strains. Additionally, we successfully detected a RIF-resistant strain with mutations at codon 531 of the *rpo*B gene in Zeptometrix verification panel. Our isothermal mutation detection system, relying on dual probes exhibits a versatile approach. With the capability to identify mutations without prior knowledge of their specific mutation direction, our dual-probe method shows significant promise for applications in drug resistance nucleic acid testing, particularly in resource-limited settings.

## Introduction

Mutations are broadly a result of random glitches in DNA replicating machinery which play an important role in evolution. Unfortunately, the golden era of antibiotics and non-judicious use of antibiotics, contributed to selective rise of mutations conferring drug-resistance. Modern medicine which relies on using antibiotics for curing infections is currently threatened by the emergence of antibiotic resistant strains [1, 2]. Traditionally, drug susceptibility testing (DST) methods, such as phenotypic culture-based assays and more recently molecular techniques have been employed for resistance detection. Polymerase chain reaction (PCR) based methods, such as ARMS-PCR, CLAMP-PCR, or hydrolysis probe Melting Curve Analysis, primer extension SNaPshot analysis after PCR amplification, are widely used to detect mutations in research and diagnostic applications [3, 4]. However, the need for thermocyclers limits the application of these methods. In general, many of these methods are often time-consuming, require sophisticated laboratory facilities and trained personnel, and may be expensive, making them less suitable for resource-limited settings where the burden of infectious disease is the highest. This has led to the development of many alternative technologies like isothermal nucleic acid amplification technologies for mutation detection that can be carried out at a constant temperature eliminating the need for sophisticated thermal-cycling equipment. Especially, in cases where the aim is not to quantify infection, but to confirm the presence or absence of infection and drug-resistance, the rapid turnaround time can be helpful in guiding patient treatment plans and decrease instances of unnecessary use of antibiotics. These methods could aid in early detection of drug-resistance infection spread and contribute towards fight against growing drug-resistance.

Many of the vastly researched isothermal techniques suffer from certain disadvantages that limit their application for mutation detection [5, 6]. The properties of enzymes or polymerases used in isothermal reaction along with no temperature cycling for renaturing and denaturing of DNA, lead to reduced stringency of primer annealing and extension which result in isothermal technologies tolerating mismatches or mutations in test target [7, 8]. Although researchers have demonstrated the feasibility of using isothermal amplification technology by integrating endonuclease (RNase H2 or Tth endonuclease IV), via ligation-initiation, or using self-stabilizing competitive primers for mutation detection, these novel methods require complicated ingredients [9–12]. Moreover, some of these methods are limited to detect only one mutation per reaction, and some of the methods require multiple primer sets to detect a single mutation.

Many isothermal techniques require higher temperatures to overcome mis-priming and non-specific reactions (e.g., Strand displacement amplification) or temperatures low enough for enzyme activity that it can lead to an increase in false-positive due to non-specific interactions (e.g., Nucleic acid sequence-based amplification). In some cases, multiple enzymes are needed for mutation detection which could potentially drive the cost higher. For example, helicase-dependent isothermal amplification (HDA) technology requires use of multiple enzymes since the helicase enzyme does not have sequence specific activity [10, 12–15].

Tuberculosis (TB) is one such disease, that still persists in the present due to the emerging multi-drug resistant (MDR) strains. As a major global health threat TB causes millions of new infections and deaths each year. As recent as 2020, compounded by the COVID-19 pandemic, an estimated 1.9 million deaths were attributed to tuberculosis [16]. The global efforts to fight TB are continuously being thwarted by the emerging drug-resistance strains, particularly those resistant to rifampicin (RIF) pose a significant challenge [17]. Rifampicin is a key first-line antibiotic used in the standard TB treatment regimen, and resistance to this drug combined with isoniazid resistance is often a marker of multidrug-resistant TB (MDR-TB) or extensively drug-resistant TB (XDR-TB) [18]. Prompt and accurate detection of RIF resistance (RR-TB) is crucial for guiding appropriate treatment decisions, preventing the spread of drug-resistant strains, and improving patient outcomes. Mutations commonly occurring in the 81-bp long region of the *rpo*B gene, which encodes the beta subunit of RNA polymerase, called the RIF resistance-determining region (RRDR) can lead to resistance to RIF (also referred to as RIF-resistance). Ninety percent of the RIF-resistance is linked to mutations in this key region, with most common mutations occurring at codons 516 (resulting in Asp to Val or Tyr), 526 (resulting in His to Leu or Tyr), and 531(resulting in Ser to Leu or Trp) based on *E*. *coli rpo*B gene numbering system [19, 20].

**Fig 1:**
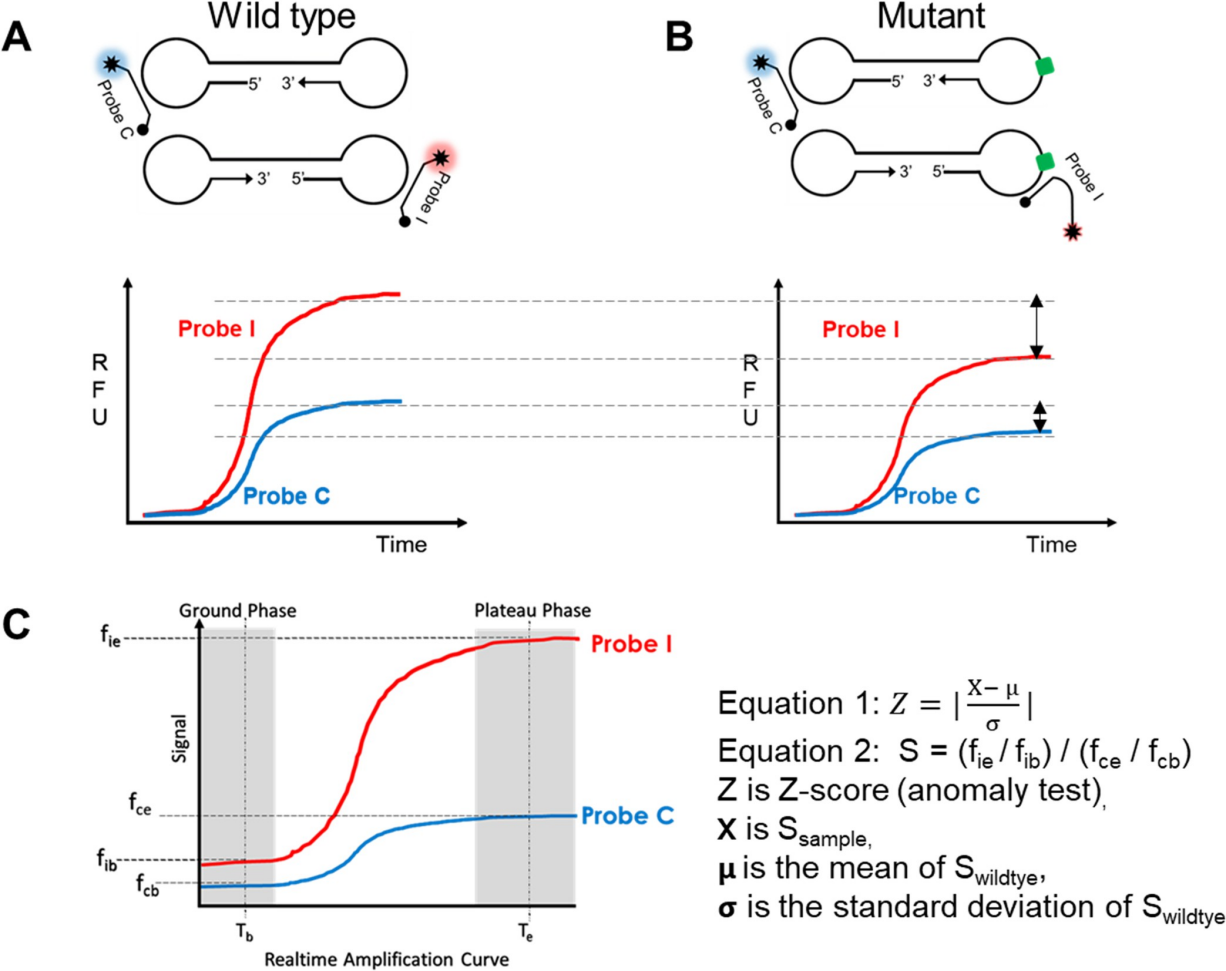
Schematic diagram of dual probe based mutation detection. (A-B) Hybridization of probes to the amplicon or intermediary amplification product obtained using isothermal amplification method like LAMP. The signals from the probes can deviate depending on the presence or absence of mutation or template concentration. Mutation is determined by deviation of calibrated signals from sample compared to that from wild type. The raw data signal is related to the amount of amplified product and hybridization efficiency between target and probe. Lower panel: an example by real time detection. WT: Wild type; Probe I: “Indicator” probe; Probe C: “Calibrator” probe. (C) A plot of dual probe detection using real-time readout in isothermal amplification. X-axis is time; Y-axis is signal; T_b_: baseline or background signal time point; T_e_: plateau phase signal end time point. Right panel: Equations for signal calibration. Z-score is used to determine presence or absence of mutation.

In this study, we established a dual probe method for mutation detection. To demonstrate it’s applicability in resource limited setting, we selected Loop mediated amplification (LAMP) to amplify the target sequence. This dual probe method design allows for use of two probes that bind to different regions of the same amplicon formed from a primer set making the assay design simple. One of the probes binds to a conserved sequence acting as a signal calibrator (Probe C) and the second probe binds to the mutation site acting as a mutation indicator (Probe I) (Fig 1). Additionally, considering signals from both the probes helps mitigate variations introduced due to reaction conditions. We also show that multiple closely located mutations can be detected using same primer set. Although the dual probe method described in this study was used in conjunction with LAMP isothermal amplification, the dual probe strategy can be applied to amplicons generated by any other isothermal technologies.

## Materials and methods

### Materials

Wild type and mutant type genomic DNA of *Mycobacterium tuberculosis* from ATCC (Cat#: 25177DQ, BAA-2237D-2, BAA-2236D-2, 35838D-2) and BEI resources (Cat#: NR-44096) were obtained for the assay. NATtrol^TM^ MTB verification panel was purchased from ZeptoMetrix (Cat# NATMTBP-C).

**Fig 2:**
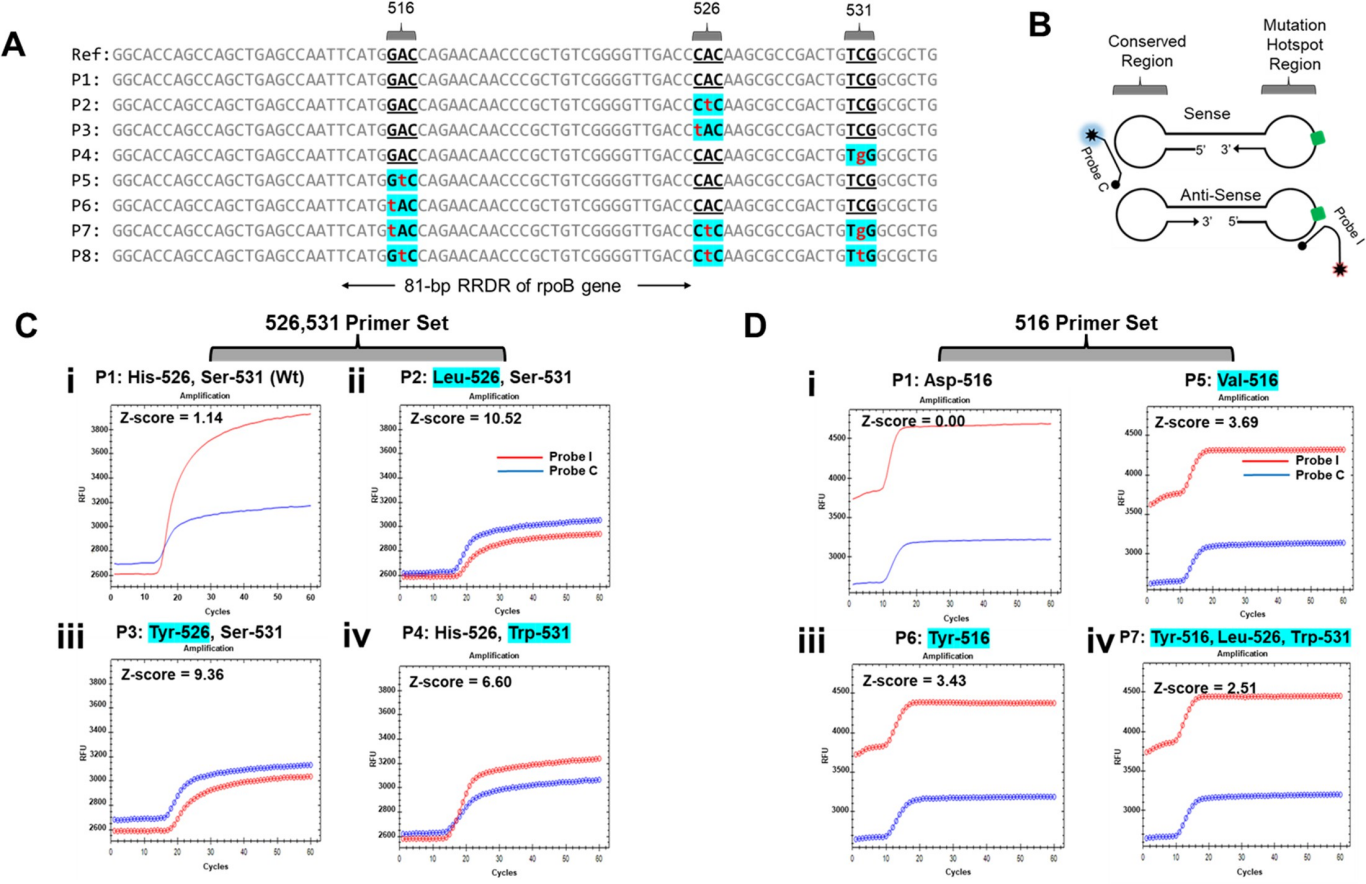
RIF-drug resistance related mutation sites and their detection by the dual probe method. (A) Sequences of plasmid inserts used for the study. The mutation sites considered in the study are highlighted in blue and the mutations are denoted in small alphabet in red. (B) Probe C and Probe I binding region RIF drug resistance detection using dual probe based strategy in LAMP. (C) Mutation detection at 526 and 531 sites using dual probe method. Fluorescence signals from the calibrator (Probe C) and indicator (Probe I) probes in presence of plasmid samples: (i) Wild type plasmid (His-526, Ser-531). (ii) P2 plasmid (Leu-526, Ser-531). (iii) P3 plasmid (Tyr-526, Ser-531). (iv) P4 plasmid (His-526, Trp-531). (D) Mutation detection at site 516 using dual probe method. Fluorescence signals from the calibrator (Probe C) and indicator (Probe I) probes in presence of plasmid samples: (i) Wild type plasmid (Asp-516). (ii) P5 plasmid (Val-516). (iii) P6 plasmid (Tyr-516). (iv) P7 plasmid (Tyr-516, Leu-526, Trp-531). X-axis is time (min), Y-axis is fluorescence signal (RFU). Z-score is labeled in each sample’s dual probe detection plot.

DNA fragments with wild type and mutation of different types and at different locations in the MTB DNA sequences were synthesized and cloned into pUC19 plasmid by GenScript (Fig 2). Concentrations of these plasmids were quantified by Bio-Rad ddPCR platform following the user guide.

### Isothermal amplification and signal collection

4X-Lyo-ready LAMP mix from Meridian BioSciences (cat#MDX097) was used for the LAMP reactions. HPLC-purified primers and probes were purchased from either IDT or Sigma Aldrich, and resuspended using TE buffer (Sigma Aldrich, cat# 93283) (S1 Table in S1 File). LAMP reaction was performed in a final 15 μL reaction mixture consisting of 1X-Lyo-ready LAMP mix, and 1.6 μM (each) of the primers FIP and BIP, 0.2 μM (each) of the primers F3 and B3, 0.4 μM (each) of primers LF and LB, 0.4 μM (each) of the calibrator probe (Probe C) and the indicator probe (Probe I), 8.0 mM MgSO_4_ and target nucleic acid template either genomic DNA or plasmids. Target amplification and real-time signal collection were carried out at 66°C for 60 mins on CFX96 Touch Real-Time PCR Detection System from Bio-Rad.

### MTB verification panel

Samples from the Zeptometrix MTB verification panel were extracted prior to LAMP assay as described in detail below: 200 μL of the wild type strain and 600 μL of the RIF-resistance strain were centrifuged at 12000 rpm for 5 minutes. The pellet obtained after discarding the supernatant was resuspended in 200 μL TE buffer, and then heated at 95°C for 30 minutes and cooled to room temperature. Genomic DNA from the heat lysed ZeptoMetrix MTB verification panel samples was purified and eluted in 40 μL TE using Oligo Clean & Concentrator (Cat# D4061, Zymo Research, CA, USA). 5 μL of extracted DNA was used as input for the LAMP assay.

Genomic DNA isolated from 4 wild type and 1 RIF-resistant clinical isolates of Mycobacterium tuberculosis purchased from ATCC and BEI were diluted in TE buffer. The samples were diluted based on concentration provided by the vendor to obtain an approximate concentration of 1332 cp/rxn in the final LAMP reaction. 5 μL of diluted DNA samples were used as input for the LAMP assay.

### Statistical analysis

Realtime fluorescence signals obtained were divided into different regions as shown in Fig 1C. Z-scores calculated based on Eqs 1 and 2 were used to determine the sample type as wild type or mutant.

$$Z=|\frac{X-\mu }{\sigma }|$$ Eq (1)

where,

X: S_sample_

μ: S_wt_

σ: standard deviation of S_wt_

the values of S for the wild type reference and test samples are calculated using the following equation,

$$S=\left(f_{ie}\;/\;f_{ib}\right)\;/\;\left(f_{ce}\;/\;f_{cb}\right)$$ Eq (2)

where,

f_cb_: Baseline signal of “calibrator” probe

f_ce_: End point signal of “calibrator” probe

f_ib_: Baseline signal of “indicator” probe

f_ie_: End point signal of “indicator” probe

RFU values at 10 minutes were designated as baseline signals for both the probes (f_cb_ and f_ib_) since amplification was observed after 10 minutes in most cases. As for the endpoint signals for both the probes (f_ce_ and f_ie_), RFU values at 40 minutes were selected since most signals reached plateau after 30 minutes.

A threshold value: Z_T_ for each primer set, is determined by the S values from the wild type. A Z-score greater than the threshold value (e.g., Z > Z_T_) indicates presence of mutation and a Z-score value is less than or equal to the threshold value (e.g., Z ≤ Z_T_) indicates the absence of mutation.

## Results

### Dual probe method principle and statistical model for mutation detection

Isothermal techniques like LAMP that despite having rapid processivity encounter the disadvantage of being unable to provide single nucleotide differentiation due to low-stringency for primer annealing at defined temperature, typically below 68°C [21]. Anderson et al in their mini review have highlighted some attempts at designing quantitative LAMP assay by using different primer modification techniques [21]. However, many methods do not address the variations in signals caused by background signals, reaction conditions, and low target copy number [22]. To address these signal variations, we propose a dual probe method for mutation detection using isothermal amplification technology. In this method (Fig 1A and 1B), one probe detects a conserved region of the target nucleic acid and acts as a calibrator (Probe C), the other probe binds the region of mutation in the target nucleic acid sequence and acts as the mutation indicator (Probe I). Both Probe C and Probe I are designed such that they are complimentary to the wild type sequence. In theory, a probe complimentary to the wild type sequence, like Probe I, would give reduced or no signal in presence of the mutated sequence. However, such signal reduction or loss could be attributed to many other factors such as reaction inhibition, background noise, or a failed reaction. As Probe C binds to the same amplicon as Probe I, the signal from Probe C would be similarly affected by the reaction conditions as Probe I. This makes Probe C the ideal internal signal calibrator, to address the variations in signals due to various factors.

Since the dual probe method, takes into consideration the fluorescence signals of both the calibrator and indicator probe to determine the presence or absence of the mutation in the sample (Fig 1A and 1B), a statistical method model was used to process the signals. To choose a proper statistical model, we first sought to consider all the possible signal relationships between Probe I and Probe C for mutation detection (See details in S1 and S2 Figs). To ensure the mutation detection is not affected by phenomena such as reaction inhibition, background noise, and guided by the shape of signal curves between Probe I and Probe C (Figs 1A, 1B and S1), our statistical model considers both the baseline signals in the ground phase and the end point signals in the plateau phase of the amplification curve. Probe I and Probe C end-point signals are normalized by their own ground phase and endpoint signals (f_ie_ / f_ib_ and f_ce_ / f_cb_ in Fig 1C) before we calculate the value of S (the ratio of Probe I to Probe C, Equation 2 in Methods). Z-score which measures how many standard deviations S_sample_ (test sample) is away from the mean of S_wt_ (wildtype samples) was finally calculated. A threshold or cut-off value of Z_T_ for Z-score was determined using reference wildtype cohort samples, any sample with Z-score greater than Z_T_ will be considered as outliers or be called mutants (see definitions and equations in Methods).

### Dual probe method doesn’t compromise the sensitivity of the assay

To test the feasibility of our dual probe mutation detection method, we designed LAMP primers to amplify RRDR region of *rpo*B, and two molecular beacon probes with Probe C binding a conserved region and Probe I binding the mutation hotspot region in the amplicon. Generally, in LAMP systems, the probes and loop primer pair of LAMP are designed in the same single-stranded region in dumbbell amplicon structure (Fig 1A), which could exhibit a competitive hybridization relationship in reaction. Introducing a second probe into LAMP reaction could potentially interfere with the efficiency of the conventional one probe-based LAMP assay. To evaluate this, different concentrations of the reference ATCC genomic DNA were tested against each probe individually and together. As seen in S3 Fig, the target detection efficiency of the assay in presence of either only calibrator probe or only indicator probe or in presence of both the calibrator and the indicator probe is similar with assay sensitivity at ~100 copies/reaction (cps/rxn).

Another potential drawback in using probe-based detection is that the fluorescence signal can be affected by the template concentration [23]. To evaluate if the internal calibration Probe C used in our dual probe method can help overcome this drawback, we investigated signal amplification curves with wild type and mutant sequences (S4 Fig) at concentrations 37–4000 cps/rxn. As expected, fluorescence signal variations occurred across different reactions and sample concentrations. But more excitingly, both the calibrator and indicator probes signals were affected proportionally (S4 Fig). In summary, using the calibrator probe signal as ruler for the indicator probe makes the system robust and independent of template concentration and other system variables.

Also, many probe-based detection methods designed to detect specific mutation sometimes cannot distinguish between wild type and other substitution mutations at the same site since the type of substitution can affect the probe binding efficiency and in turn the amplification signal [24]. For instance, RIF drug resistance is linked to both Ser531Leu and Ser531Trp mutations. Probes designed to detect the more commonly occurring Ser531Leu might fail to distinguish between wild type and Ser531Trp. To evaluate if the dual probe method can be used to detect different mutations irrespective of the type of substitutions, we investigated contrived samples with different mutations Ser531Leu and Ser531Trp. The results in S4 Fig show that dual probe method could distinguish both Ser531Leu and Ser531Trp mutations from wild type.

### Dual probe method performance in contrived samples

To systematically evaluate the MTB RIF drug resistance detection capability of the dual probe method, we obtained eight plasmids with inserts containing major mutations at the 516, 526 and 531 codons of the RRDR regions of *rpo*B (Fig 2A). Two primer sets for LAMP were designed: one primer set for 526, 531 mutation detection, the other set for 516 mutation detection. A reference wild type training data set (N = 12 for both 526, 531 primer set, and 516 primer set) to determine Z-score threshold (Z_T_) was obtained using MTB ATCC DNA (S2 and S3 Tables in S1 File). Plasmids carrying the wild type sequence and different types and combinations of mutations were then tested with either the 526, 531 primer set (N = 47) or the 516 primer set (N = 80). The fluorescence values at baseline and endpoint for both the calibrator and indicator probes used to calculate the Z-score values for the samples are given in S4 and S5 Tables in S1 File.

Fig 2B shows the binding sites for Probe C and Probe I on the sense and anti-sense strands of the amplicon. Fig 2C and 2D show example amplification curves of some of the samples tested (samples in bold in S4 and S5 Tables in S1 File). In the 526, 531 primer set shown in Fig 2C, consistent fluorescence signals from Probe C were observed irrespective of presence or absence of mutations. This consistency in Probe C signal (blue amplification curves) is as expected since it is designed to bind a conserved sequence on the amplicon and remains unaffected by the wild type or mutant sequences. The fluorescence sequence from Probe I on the other hand is fully complementary to the wildtype sequence (Fig 2B). The changes in Probe I signal intensities (red amplification curves) observed between wild type and mutation sequence can be attributed to the differential binding efficiency of Probe I to the target region (Fig 2B and 2C). The results also show that mutations located in close proximity (example codon 526 and 531) could be detected using same set of primers and probes. Similar trends for Probe C and Probe I signals could be observed in 516 primer set as seen Fig 2D.

**Fig 3:**
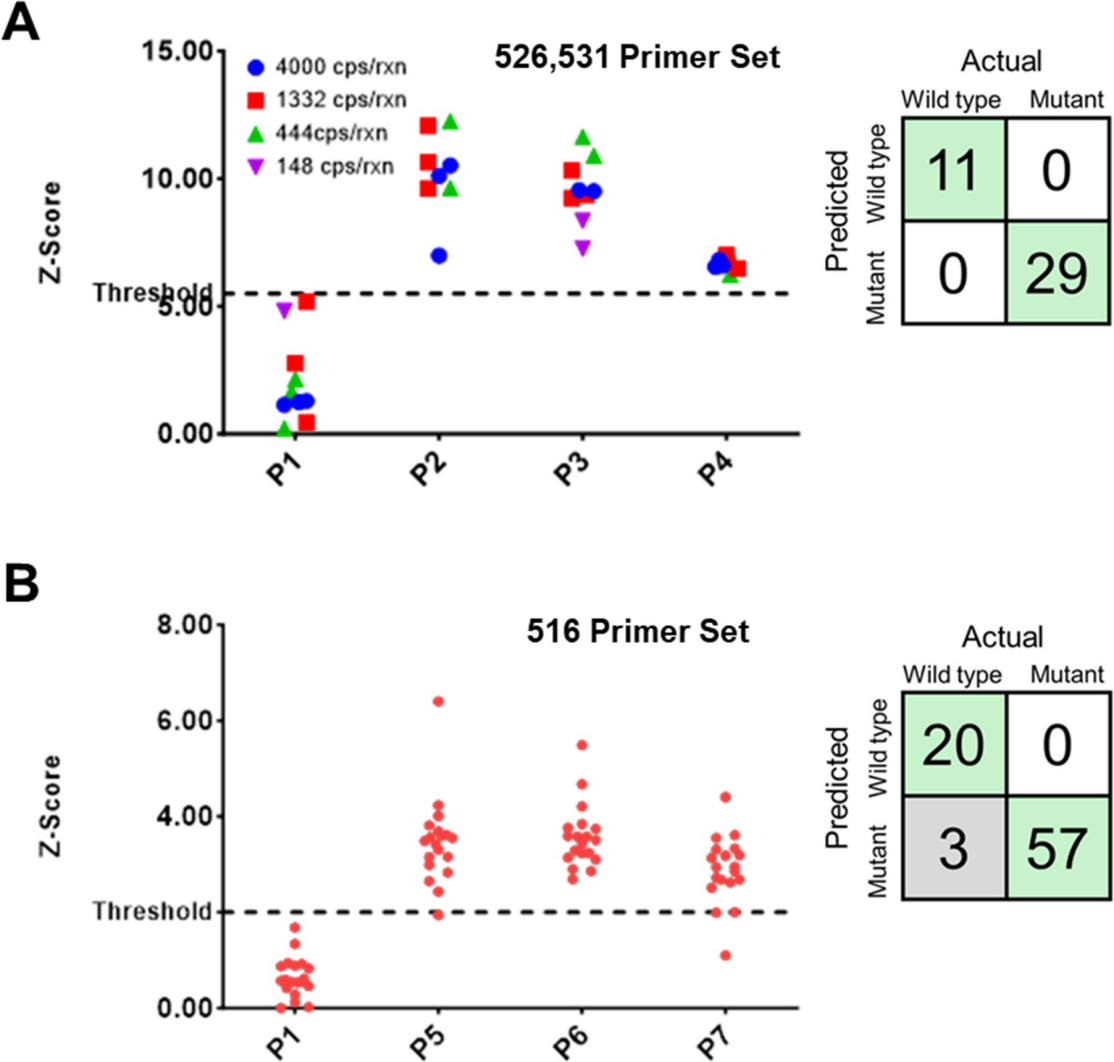
Dual probe based mutation detection performance on contrived samples. (A) Scatter plot of Z-score of 40 valid contrived samples of different genotypes at different concentrations for detection of mutations at sites 526 and 531 using the 526, 531 primer set. Initial sample concentration in reaction is labeled using different shapes. Dashed line Z-score = 5.5 is a threshold. The 2X2 table showing 100% accuracy in determining the sample type. (B) Scatter plot of Z-score of 80 valid contrived samples of different genotypes at different concentrations for detection of mutations at site 516 using the 516 primer set. Dashed line Z-score = 2 is a threshold. The 2X2 table shows 100% accuracy for wild type detection and 95% accuracy for mutation detection.

The 47 samples tested with the 526, 531 primer set were comprised of different concentrations of wild type sequences and RIF mutations at codons 526 and 531. Among the 47 samples tested, 7 samples were considered invalid due to failed reactions with template concentrations falling below the reaction sensitivity, as indicated in S2 Fig. Using the Z_T_ of 5.5 obtained from training data set, the remaining 40 samples were differentiated into wild type and mutant (Fig 3A). 11 out of 11 were correctly identified as wild type and all the 29 samples with mutated sequences were identified with 100% accuracy irrespective of the concentration of the plasmid in the sample (Fig 3A 2X2 table).

Since the template concentration did not influence the sample identification, samples at a constant concentration of 1000 cps/rxn were tested for the 516 primer set. A Z_T_ value of 2 was obtained from the training dataset using the 516 primer set. 80 samples comprising of 20 wild type sequences and 60 sequences with RIF mutations at codon 516 were tested using the assay (Fig 3B). With a threshold of 2, 20 out of 20 were correctly identified as wild type and 57 out of 60 samples with mutated sequences were identified with 96% accuracy (Fig 3B 2X2 table).

### Dual probe method evaluation with MTB verification panel

**Fig 4:**
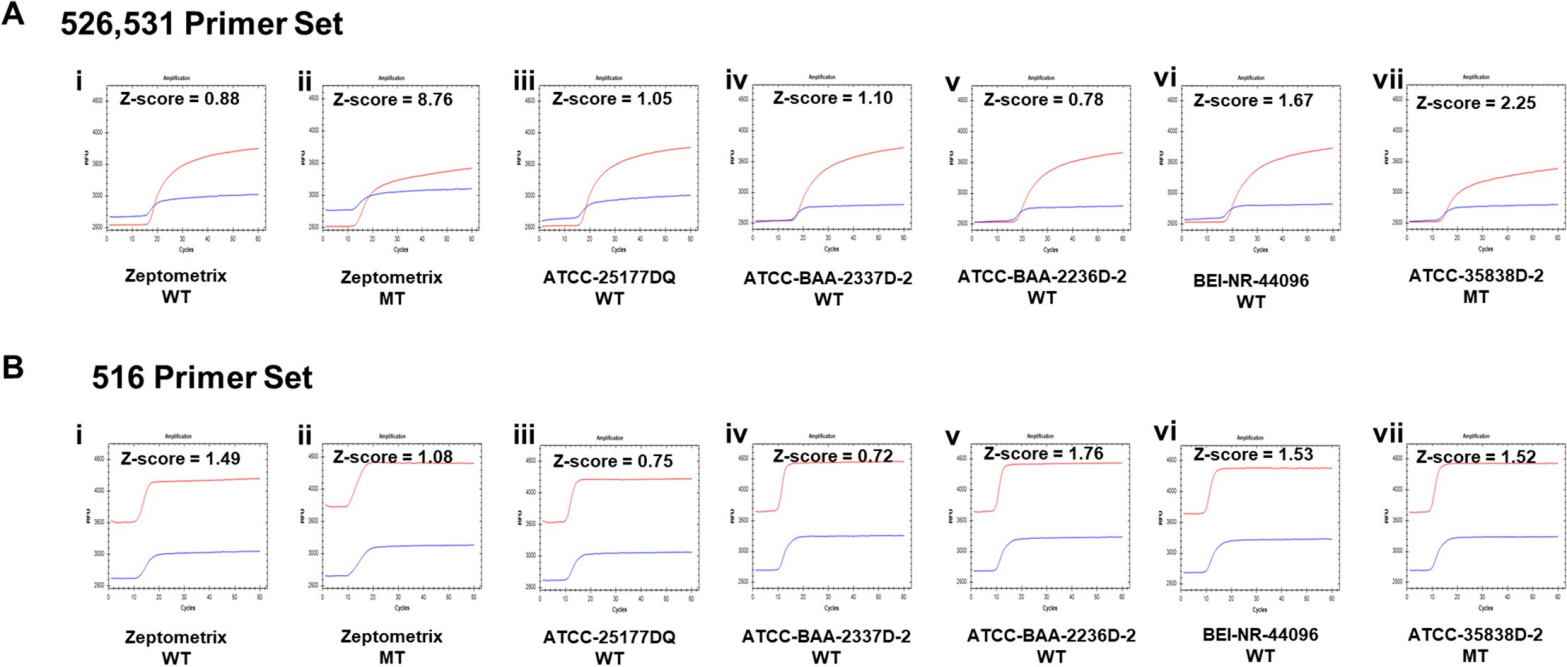
Mutation detection in MTB verification panel. (A) Fluorescence signals from the calibrator (Probe C, blue) and indicator (Probe I, red) probes (designed to detect mutations at sites 526 and 531). (B) Fluorescence signals from the calibrator (Probe C, blue) and indicator (Probe I, red) probes (designed to detect mutations at site 516). (i) Zeptometrix Wild type strain, (ii) Zeptometrix RIF-resistance strain carrying Ser531Leu change, (iii) Wild type strain (ATCC-25177DQ), (iv) Wild type strain (ATCC-BAA-2337D-2), (v) Wild type strain (ATCC-BAA-2336D-2), (vi) Wild type strain (BEI-NR-44096), (vii) Mutant type strain (ATCC-35838D-2) carrying single mutation at codon 531 (TCG to TTG). X-axis is time (min), Y-axis is fluorescence signal (RFU). Z-score is labeled in each sample’s dual-probe detection plot. Clinical strains ID are labeled below each plot.

To further evaluate the dual probe method for MTB drug resistance detection, we obtained wild type and mutant type genomic DNA from ATCC, BEI and MTB verification panel from Zeptometrix. The wild type samples from ATCC and BEI were genomic DNA extracted from clinical isolates, whereas the Rif- mutation carrying sample from ATCC was an *in vitro* mutant of a wild type clinical isolate (codon531, TCG to TTG). The Zeptometrix samples on the other hand are formulated with purified, intact bacterial cells. After extraction and purification, wildtype and mutant strains’ DNAs from Zeptometrix and the diluted DNA from ATCC and BEI were tested by our 526,531 LAMP primer set and 516 LAMP primer set. All wild type strains exhibited Z-score below the threshold values for mutation detection (Z_T526,531_ = 5.5, Z_T516_ = 2), indicating these strains are rifampicin-sensitive (Fig 4A i, iii, iv-vi and Fig 4B i, iii, iv-vi.).

Please note that one Zeptometrix strain in Fig 4A ii, shows a Z-score value of 8.76 in 526, 531 primer set, exceeding the threshold value of 5.5, indicating rifampicin resistance. This finding aligns with the presence of Ser531Leu change in this strain, as reported by Zeptometrix. Interestingly, in the RIF-resistant strain from ATCC, while the probe I signal decreased noticeably in the 526, 531 primer set (Fig 4A vii, red curve), the Z-score was 2.25, which is below our predefined threshold of 5.5 with the current limited training and validation dataset. Consequently, we failed to make a mutation call for this specific single mutation change at codon 531 (TCG to TTG). On the other hand, at codon 516 region (Fig 4B), the signal of probe I of these two drug resistance strains shows a trend similar to all other drug sensitive strains, indicating absence of mutation at codon 516. This agrees with the absence of any mutation at the 516 site in the RRDR regions of *rpo*B in the Zeptometrix and the ATCC RIF-resistant strain.

## Discussions

Many assays designed for drug resistance related mutation detection can be relatively expensive, require sophisticated instruments, are impacted by inhibitors in sample resulting in false-negative results [25, 26]. This makes using such methods less than ideal for high threat regions. The dual probe method proposed here has simple ingredients, is affordable, rapid, doesn’t require sophisticated instruments. The incorporation of a probe as an internal control serves to mitigate signal variations from template concentration or system variables (e.g., instrument fluorescence readings) or reaction conditions (e.g., inhibitions from sample matrix). This, in turn, contributes to a reduction in both false positive and false negative rates. Although the Bio-Rad CX96 real-time machine and the liquid reagent format are used for this proof-of concept study, the reaction was carried out at a constant temperature which could be performed on any instrument designed for isothermal amplification applications and the liquid reagents could be replaced with the available dry format to overcome any cold chain storage hurdles.

Many systems require the use of multiple probes in order to detect each type of mutation, which is necessary when studying different substitutions at given mutation site. Also, many commercially available nucleic acid amplification tests (NAATs) such as Xpert Mycobacterium tuberculosis (MTB)/rifampicin (RIF) assay, GenoType MTBDRplus line probe assay (LPA), and TrueNAT MTB/MTBplus/MTB-RIF, use different approaches to target mutations known to be associated with Rif-drug resistance. Xpert, nested-real time PCR based assay for example uses the difference in Ct values between multiple probes that cover the RRDR region of rpoB for Rif-resistance detection [27, 28]. Our dual probe method uses isothermal amplification method with probes designed for both the mutation region and a conserved region outside the *rpo*B gene on the same amplicon. This method with further validation could potentially also detect the presence of non-targeted mutations in presence of signals from the control probe and absence of signal from the indicator probe (S1(vi) Fig). So, in cases where mutation irrespective of the type of the substitution or an emerging new mutation leading to drug resistance are encountered, the current dual probe method is a simpler design that allows for rapid mutation detection.

Many point-of-care colorimetric based LAMP reactions have already been reported. Our method, however, overcomes one of the major drawbacks related to specificity encountered in these LAMP reactions [29–31]. First, using molecular beacon ensures target specificity. Second, the incorporation of the calibrator probe ensures that the mutation detection is not affected by template concentration and other system variables. The probes in our method detect target regions on the same amplicon allowing us to use single primer set for amplification. Using this assay design, we were able to identify mutations at codon 516, 526 and 531, that cover more than 90% RIF related drug resistance cases reported, with 100% accuracy for mutations at 526 and 531 sites and 95% accuracy for mutations at site 516 in the evaluated contrived sample set.

All the Rif-susceptible strains from Zeptometrix, ATCC and BEI were identified as wild type with 100% accuracy using the dual probe method. The Rif-resistant strain from Zeptometrix, having a higher Z-score than the threshold value (Z_T526,531_ = 5.5) was also identified accurately as mutant. The method was, however, unable to identify the Rif-resistant strain from ATCC. When investigating this ATCC Rif-resistant strain, we observed that our dual probe method’s training and validation mutation panel using contrived samples does not encompass the specific single mutation (codon 531: TCG to TTG) present in the ATCC drug-resistant strain. As a result, the statistical model for distinguishing mutations may not be adequately trained to accurately detect this variation. It may be necessary to modify probe I to enhance signal change for all potential mutation directions to avoid false negative results.

Also, the lack of availability of more non-infectious Rif-resistant *Mycobacterium tuberculosis* genomic DNA samples with Rif-mutations at different sites, limits the sample size for testing and doesn’t allow to fully explore the performance of the dual probe method. The results from the contrived sample do help demonstrate the potential of the dual probe method in detecting single nucleotide polymorphism which is often associated with drug resistance. The limited clinical sample along with the constrained nature of our synthetic DNA fragments that were designed to encompass only regions and mutations relevant to our assay design, restricts the comparison of our technology to commercially available tests. Hereafter, increasing the sample size to encompass more clinical strains to increase our training dataset and mutant samples with varying mutations in the RRDR sequence would help further optimize the assay design.

In conclusion, the dual probe technology holds great promise for mutation detection. Specifically, when used with isothermal nucleic acid amplification, its rapidity, affordability, and suitability for resource-limited settings make it an attractive alternative to conventional PCR methods. To fully leverage its potential in guiding appropriate treatment decisions, limiting the spread of resistance, and enhancing patient outcomes in infectious disease control programs, further clinical studies are needed to explore the performance of this technology in timely detection of drug-resistant strains. It is also essential to note that the dual probe concept has its limitations. It may not be able to differentiate drug-resistance conferring mutations from silent mutations at the same locations [32, 33], and it may not be able determine whether the samples are a heterozygous pool or homozygous pool.

## Supporting information

S1 File S1 to S5 Tables. (DOCX)

S1 Fig Schematic representation of possible results using the dual-probe method (using real-time readout as an example). (i) Realtime amplification curve of Wild type control sample. (ii) A mutant sample in which calibrator probe signal does not change while indicator probe signal dramatically drops due to effects of mutations on the indicator probe-target hybridization efficiency. (iii) A wild type in which signals from calibrator probe and indicator probe synchronously decrease. This pattern might result from relatively low concentration of the sample, or high non-specific product, low reaction activity. The sample type identification is not affected since, calibrated score does not change. (iv) A mutant in which calibrator probe signal increases while indicator probe signal does not change. Calibrated score changes. (v) Invalid as there is no signal from endogenous reference probe (i.e., calibrator probe). The indicator probe signal could be a result of some cross contamination or non-specific amplification. (vi) A mutant that calibrator probe shows presence of target, no signal from mutation indicator probe. (vii) Negative or Invalid as neither of probes has signals. This result might be due to expired reagents, sample interference, or low concentration or absence of target nucleic acid.
(TIF)

S2 Fig Decision flow chart for sample type determination. (TIF)

S3 Fig Effect of presence of two probes on efficiency of assay. (A) Fluorescence signals from the calibrator (Probe C) probe (designed to detect mutations at sites 526 and 531) in presence of MTB reference wild type genomic DNA. (B) Fluorescence signals from the indicator (Probe I) probe (designed to detect mutations at sites 526 and 531) in presence of contrived plasmid samples. The sensitivity of the assay lies between 100 and 50 cps/rxn irrespective of the presence of single or both probes. X-axis is time (min), Y-axis is fluorescence signal (RFU).
(TIF)

S4 Fig Effect of template concentration on assay efficiency. (A) Fluorescence signals from the calibrator (Probe C) probe (designed to detect mutations at sites 526 and 531) in presence of 37–1000 cps/rxn of MTB reference wild type genomic DNA. (B) Fluorescence signals from the calibrator (Probe C) and indicator (Probe I) probes (designed to detect mutations at sites 526 and 531) in presence of contrived plasmid samples. The probe signals are similar irrespective of the template concentration. X-axis is time (min), Y-axis is fluorescence signal (RFU).
(TIF)
