# Correction: A novel dual probe-based method for mutation detection using isothermal amplification

**DOI:** 10.1371/journal.pone.0355622

**Published:** 2026-08-06

**Authors:** Nidhi Nandu, Michael Miller, Yanhong Tong, Zhi-xiang Lu

In Fig 3, there is an error in the 2x2 table of panel B. Please see the correct Fig 3 here.

**Fig 3:**
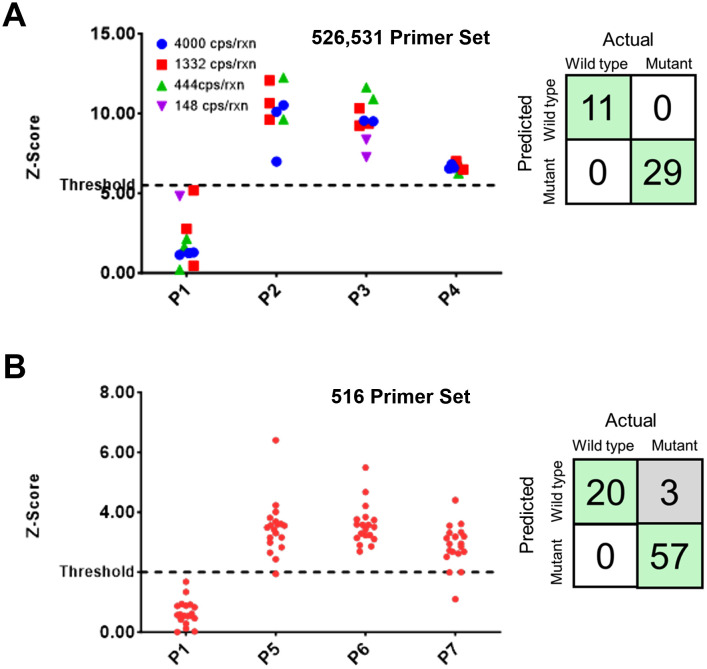
Dual probe based mutation detection performance on contrived samples. (A) Scatter plot of Z-score of 40 valid contrived samples of different genotypes at different concentrations for detection of mutations at sites 526 and 531 using the 526, 531 primer set. Initial sample concentration in reaction is labeled using different shapes. Dashed line Z-score = 5.5 is a threshold. The 2X2 table showing 100% accuracy in determining the sample type. (B) Scatter plot of Z-score of 80 valid contrived samples of different genotypes at different concentrations for detection of mutations at site 516 using the 516 primer set. Dashed line Z-score = 2 is a threshold. The 2X2 table shows 100% accuracy for wild type detection and 95% accuracy for mutation detection.
