# Supplementary material for: A novel dual probe-based method for mutation detection using isothermal amplification
Source: PLoS One. 2024 Oct 22;19(10):e0309541. doi: 10.1371/journal.pone.0309541 (PMC11495626; doi:10.1371/journal.pone.0309541)
Supplement: S1 File — (DOCX) [file pone.0309541.s001.docx]

**A Novel Dual Probe-based Method for Mutation Detection using Isothermal Amplification**

Nidhi Nandu^1^, Michael Miller^1^, Yanhong Tong^1*^ and Zhi-xiang Lu^1*^

^1^Revvity, Inc., Waltham, MA, USA

*Corresponding authors

E-mail: Zhixiang.Lu@revvity.com (ZL)

E-mail: Yanhong.Tong@revvity.com (YT)

**Keywords:** Dual Probe, mutation detection, LAMP, drug resistance, Rifampicin, MTB

**Supporting Information**

**S1 Table.** **Sequences of primers and probes**

| **Oligo Name** | **Sequences (5’ – 3’)** |
| --- | --- |
| F3_526,531_ | AGCGGATGACCACCCAG |
| B3_526,531_ | TGCACGTCGCGGACCT |
| FIP_526,531_ | CTTGATCGCGGCGACCACCGGACGTGGAGGCGATCACA |
| BIP_526,531_ | CAGAACAACCCGCTGTCGGCACGCTCACGTGACAGACC |
| LF_526,531_ | GCCGGATGTTGATCAACG |
| LB_526,531_ | CCACAAGCGCCGACTG |
| Probe C_526,531_ | FAM-CGCGAGCCGGATGTTGATCAACGTCTGCTCGCG-BHQ1 |
| Probe I_526,531_ | Cy5-CGCGAGACC[+C][+A][+C]AAGCGCCGACTG[+T][+C][+G]GCGCTCGCG-BHQ2* |
| F3_516_ | AGCGGATGACCACCCAG |
| B3_516_ | CGCTCACGTGACAGACCG |
| FIP_516_ | CTTGATCGCGGCGACCACCGGACGTGGAGGCGATCACA |
| BIP_516_ | GTTCTTCGGCACCAGCCAGCTGCCGACAGTCGGCGCTT |
| LF_516_ | GATGTTGATCAACGTCTGCG |
| LB_516_ | GAGCCAATTCATGGACCAGAA |
| Probe C_516_ | Cy5-CGCGAGCCGGATGTTGATCAACGTCGCG-BHQ2 |
| Probe I_516_ | FAM-CGCGACCAATTCATG[+G][+A][+C]CAGAACAACTCGCG-BHQ1* |

*: [+] is LNA modified base.

**S2 Table.** **S_wt,526,531_ calculation using *Mycobacterium tuberculosis* genomic DNA.**

| **Standards** | **f_ib_** | **f_ie_** | **f_cb_** | **f_ce_** | ***S_wt,526,531_= (f_ie_ / f_ib_) / (f_ce_ / f_cb_)** |
| --- | --- | --- | --- | --- | --- |
| MTB-gDNA | 2664 | 3621 | 2891 | 3323 | 1.18 |
| MTB-gDNA | 2668 | 3927 | 2903 | 3390 | 1.26 |
| MTB-gDNA | 2657 | 3658 | 2940 | 3401 | 1.19 |
| MTB-gDNA | 2662 | 3826 | 2842 | 3295 | 1.24 |
| MTB-gDNA | 2675 | 3812 | 2822 | 3260 | 1.23 |
| MTB-gDNA | 2650 | 3666 | 2910 | 3354 | 1.20 |
| MTB-gDNA | 2649 | 3855 | 2785 | 3237 | 1.25 |
| MTB-gDNA | 2650 | 3915 | 2796 | 3282 | 1.26 |
| MTB-gDNA | 2632 | 3699 | 2761 | 3212 | 1.21 |
| MTB-gDNA | 2639 | 3743 | 2762 | 3170 | 1.24 |
| MTB-gDNA | 2633 | 3615 | 2746 | 3153 | 1.20 |
| MTB-gDNA | 2634 | 3627 | 2779 | 3245 | 1.18 |

*Note: Mean (µ) S_wt,526,531_ =1.22, Standard Deviation (σ) S_wt,526,531_ =0.03

**S3 Table.** **S_wt,516_ calculation using *Mycobacterium tuberculosis* genomic DNA.**

| **Standards** | **f_ib_** | **f_ie_** | **f_cb_** | **f_ce_** | ***S_wt,516_= (f_ie_ / f_ib_) / (f_ce_ / f_cb_)** |
| --- | --- | --- | --- | --- | --- |
| MTB-gDNA | 3771 | 4603 | 2679 | 3204 | 1.02 |
| MTB-gDNA | 3739 | 4430 | 2697 | 3178 | 1.01 |
| MTB-gDNA | 3707 | 4413 | 2669 | 3131 | 1.01 |
| MTB-gDNA | 3625 | 4261 | 2667 | 3111 | 1.01 |
| MTB-gDNA | 3585 | 4295 | 2652 | 3105 | 1.02 |
| MTB-gDNA | 3614 | 4274 | 2660 | 3106 | 1.01 |
| MTB-gDNA | 3807 | 4484 | 2678 | 3136 | 1.01 |
| MTB-gDNA | 3554 | 4161 | 2659 | 3084 | 1.01 |
| MTB-gDNA | 3777 | 4353 | 2701 | 3130 | 0.99 |
| MTB-gDNA | 3677 | 4292 | 2684 | 3137 | 1.00 |
| MTB-gDNA | 3858 | 4424 | 2689 | 3160 | 0.98 |
| MTB-gDNA | 3694 | 4281 | 2684 | 3113 | 1.00 |

*Note: Mean (µ) S_wt,516_ =1.01, Standard Deviation (σ) S_wt,516_=0.01

**S4 Table.** **Z-score for the 47 samples tested using the dual-probe method.** (Mutations at 526 and 531) (Data in bold was used to create the example amplification curves in Fig 2C)

| **Plasmid-ID** | **Concentration (cps/rxn)** | **f_ib_** | **f_ie_** | **f_cb_** | **f_ce_** | **S_s_= (f_ie_ / f_ib_)/ (f_ce_ / f_cb_)** | **Z-Score** |
| --- | --- | --- | --- | --- | --- | --- | --- |
| P1 | 4000 | 2615 | 3760 | 2705 | 3101 | 1.25 | 0.49 |
| **P1** | **4000** | **2608** | **3833** | **2702** | **3127** | **1.27** | **1.14** |
| P1 | 4000 | 2591 | 3593 | 2727 | 3128 | 1.21 | 1.29 |
| P1 | 1332 | 2585 | 3597 | 2695 | 3100 | 1.21 | 1.25 |
| P1 | 1332 | 2572 | 3724 | 2646 | 3114 | 1.23 | 0.46 |
| P1 | 1332 | 2562 | 3517 | 2608 | 3057 | 1.17 | 2.77 |
| P1 | 444 | 2572 | 3348 | 2621 | 3072 | 1.11 | 5.18 |
| P1 | 444 | 2580 | 3643 | 2696 | 3080 | 1.24 | 0.22 |
| P1 | 444 | 2583 | 3596 | 2642 | 3065 | 1.20 | 1.65 |
| P1 | 148 | 2589 | 3523 | 2684 | 3075 | 1.19 | 2.14 |
| P1 | 148 | 2553 | 3288 | 2587 | 2976 | 1.12 | 4.81 |
| P2 | 4000 | 2593 | 2931 | 2612 | 2997 | 0.99 | 10.17 |
| **P2** | **4000** | **2590** | **2906** | **2621** | **3012** | **0.98** | **10.52** |
| P2 | 4000 | 2594 | 3191 | 2648 | 3059 | 1.07 | 6.99 |
| P2 | 1332 | 2580 | 2933 | 2628 | 3028 | 0.99 | 10.11 |
| P2 | 1332 | 2583 | 2958 | 2629 | 3013 | 1.00 | 9.62 |
| P2 | 1332 | 2595 | 2819 | 2660 | 3086 | 0.94 | 12.08 |
| P2 | 444 | 2586 | 2853 | 2632 | 2984 | 0.97 | 10.65 |
| P2 | 444 | 2582 | 3010 | 2637 | 3079 | 1.00 | 9.64 |
| P2 | 444 | 2588 | 2790 | 2642 | 3055 | 0.93 | 12.25 |
| P2 | 148 | 2578 | 2581 | 2637 | 2650 | - | Invalid * |
| P2 | 148 | 2575 | 2575 | 2617 | 2635 | - | Invalid * |
| P2 | 148 | 2581 | 2574 | 2636 | 2644 | - | Invalid * |
| P3 | 4000 | 2590 | 2991 | 2608 | 3006 | 1.00 | 9.50 |
| P3 | 4000 | 2589 | 3003 | 2646 | 3066 | 1.00 | 9.51 |
| P3 | 4000 | 2649 | 3059 | 2988 | 3115 | - | Invalid* |
| P3 | 1332 | 2588 | 3016 | 2642 | 3077 | 1.00 | 9.55 |
| **P3** | **1332** | **2591** | **2992** | **2691** | **3091** | **1.01** | **9.36** |
| P3 | 1332 | 2586 | 3042 | 2640 | 3080 | 1.01 | 9.24 |
| P3 | 444 | 2594 | 2922 | 2666 | 3062 | 0.98 | 10.33 |
| P3 | 444 | 2578 | 2847 | 2647 | 3084 | 0.95 | 11.64 |
| P3 | 444 | 2584 | 2904 | 2655 | 3087 | 0.97 | 10.90 |
| P3 | 148 | 2590 | 2591 | 2773 | 2788 | 0.99 | 9.77 |
| P3 | 148 | 2571 | 3060 | 2626 | 3033 | 1.03 | 8.36 |
| P3 | 148 | 2582 | 3017 | 2854 | 3150 | 1.06 | 7.26 |
| P4 | 4000 | 2601 | 3233 | 2622 | 3022 | 1.08 | 6.48 |
| P4 | 4000 | 2599 | 3238 | 2647 | 3064 | 1.08 | 6.55 |
| P4 | 4000 | 2585 | 3208 | 2636 | 3059 | 1.07 | 6.82 |
| **P4** | **1332** | **2582** | **3190** | **2629** | **3022** | **1.08** | **6.60** |
| P4 | 1332 | 2585 | 3216 | 2629 | 3072 | 1.06 | 7.01 |
| P4 | 1332 | 2587 | 3239 | 2644 | 3086 | 1.07 | 6.69 |
| P4 | 444 | 2587 | 3262 | 2644 | 3094 | 1.08 | 6.49 |
| P4 | 444 | 2577 | 3206 | 2633 | 3050 | 1.07 | 6.66 |
| P4 | 444 | 2590 | 3267 | 2663 | 3100 | 1.08 | 6.25 |
| P4 | 148 | 2578 | 2577 | 2634 | 2651 | - | Invalid * |
| P4 | 148 | 2576 | 2577 | 2633 | 2654 | - | Invalid * |
| P4 | 148 | 2573 | 2568 | 2645 | 2659 | - | Invalid * |

**S5 Table.** **Z-score for the 80 samples tested using the dual-probe method.** (Mutations at 516) (Data in bold was used to create the example amplification curves in Fig 2D)

| **Plasmid-ID** | | **f_ib_** | **f_ie_** | **f_cb_** | **f_ce_** | **S_s_= (f_ie_ / f_ib_)/ (f_ce_ / f_cb_)** | **Z-Score** |
| --- | --- | --- | --- | --- | --- | --- | --- |
| P1 | | 3888 | 4734 | 2664 | 3179 | 1.02 | 1.08 |
| P1 | | 3790 | 4510 | 2648 | 3126 | 1.01 | 0.12 |
| P1 | | 3847 | 4613 | 2661 | 3149 | 1.01 | 0.54 |
| P1 | | 3867 | 4670 | 2656 | 3162 | 1.01 | 0.61 |
| P1 | | 3877 | 4688 | 2668 | 3170 | 1.02 | 0.87 |
| P1 | | 3803 | 4539 | 2653 | 3125 | 1.01 | 0.53 |
| P1 | | 3798 | 4559 | 2661 | 3138 | 1.02 | 0.92 |
| P1 | | 3811 | 4531 | 2661 | 3123 | 1.01 | 0.54 |
| P1 | | 3683 | 4383 | 2647 | 3093 | 1.02 | 0.94 |
| P1 | 3585 | | 4237 | 2626 | 3049 | 1.02 | 0.88 |
| P1 | 3872 | | 4680 | 2663 | 3174 | 1.01 | 0.57 |
| P1 | 3586 | | 4323 | 2632 | 3086 | 1.03 | 1.68 |
| P1 | 3861 | | 4609 | 2666 | 3164 | 1.01 | 0.02 |
| P1 | 3903 | | 4655 | 2672 | 3155 | 1.01 | 0.28 |
| **P1** | **3875** | | **4669** | **2687** | **3217** | **1.01** | **0.00** |
| P1 | 3862 | | 4636 | 2682 | 3181 | 1.01 | 0.46 |
| P1 | 3839 | | 4638 | 2675 | 3188 | 1.01 | 0.59 |
| P1 | 3852 | | 4614 | 2669 | 3161 | 1.01 | 0.42 |
| P1 | 3922 | | 4742 | 2679 | 3184 | 1.02 | 0.83 |
| P1 | 3732 | | 4477 | 2658 | 3115 | 1.02 | 1.34 |
| P5 | 3777 | | 4309 | 2672 | 3148 | 0.97 | 2.90 |
| P5 | 3715 | | 4225 | 2659 | 3121 | 0.97 | 2.83 |
| P5 | 3737 | | 4216 | 2658 | 3127 | 0.96 | 3.61 |
| P5 | 4110 | | 4492 | 2699 | 3198 | 0.92 | 6.40 |
| P5 | 3823 | | 4356 | 2670 | 3171 | 0.96 | 3.55 |
| P5 | 3960 | | 4543 | 2688 | 3234 | 0.95 | 4.01 |
| **P5** | **3892** | | **4439** | **2682** | **3194** | **0.96** | **3.69** |
| P5 | 3949 | | 4547 | 2699 | 3189 | 0.97 | 2.43 |
| P5 | 3912 | | 4453 | 2675 | 3184 | 0.96 | 3.81 |
| P5 | 3692 | | 4188 | 2648 | 3114 | 0.96 | 3.16 |
| P5 | 3712 | | 4203 | 2658 | 3125 | 0.96 | 3.30 |
| P5 | 3751 | | 4275 | 2663 | 3157 | 0.96 | 3.42 |
| P5 | 3705 | | 4241 | 2647 | 3118 | 0.97 | 2.65 |
| P5 | 3829 | | 4374 | 2688 | 3229 | 0.95 | 4.23 |
| P5 | 3809 | | 4360 | 2680 | 3197 | 0.96 | 3.56 |
| P5 | 3869 | | 4545 | 2676 | 3205 | 0.98 | 1.94 |
| P5 | 3760 | | 4297 | 2671 | 3157 | 0.97 | 2.99 |
| P5 | 3829 | | 4359 | 2681 | 3178 | 0.96 | 3.49 |
| P5 | 3758 | | 4286 | 2654 | 3156 | 0.96 | 3.59 |
| P5 | 3700 | | 4231 | 2652 | 3142 | 0.96 | 3.15 |
| P6 | 3891 | | 4335 | 2713 | 3187 | 0.95 | 4.41 |
| P6 | 3807 | | 4343 | 2683 | 3197 | 0.96 | 3.74 |
| P6 | 3778 | | 4268 | 2671 | 3146 | 0.96 | 3.59 |
| **P6** | **3851** | | **4378** | **2692** | **3185** | **0.96** | **3.43** |
| P6 | 3880 | | 4430 | 2694 | 3207 | 0.96 | 3.58 |
| P6 | 3892 | | 4422 | 2666 | 3166 | 0.96 | 3.76 |
| P6 | 3791 | | 4323 | 2669 | 3160 | 0.96 | 3.29 |
| P6 | 3988 | | 4498 | 2678 | 3175 | 0.95 | 4.21 |
| P6 | 3793 | | 4285 | 2670 | 3144 | 0.96 | 3.58 |
| P6 | 3982 | | 4369 | 2673 | 3139 | 0.93 | 5.49 |
| P6 | 3643 | | 4140 | 2661 | 3122 | 0.97 | 2.86 |
| P6 | 3709 | | 4194 | 2671 | 3134 | 0.96 | 3.23 |
| P6 | 3672 | | 4172 | 2656 | 3126 | 0.97 | 3.10 |
| P6 | 3823 | | 4305 | 2717 | 3187 | 0.96 | 3.51 |
| P6 | 3870 | | 4394 | 2688 | 3193 | 0.96 | 3.84 |
| P6 | 3808 | | 4337 | 2672 | 3153 | 0.97 | 3.14 |
| P6 | 4015 | | 4535 | 2714 | 3245 | 0.94 | 4.67 |
| P6 | 3762 | | 4309 | 2656 | 3133 | 0.97 | 2.69 |
| P6 | 3752 | | 4308 | 2665 | 3160 | 0.97 | 2.90 |
| P6 | 3666 | | 4168 | 2643 | 3118 | 0.96 | 3.24 |
| P6 | 3706 | | 4223 | 2665 | 3121 | 0.97 | 2.55 |
| P7 | 3667 | | 4179 | 2676 | 3112 | 0.98 | 2.00 |
| P7 | 3634 | | 4128 | 2664 | 3138 | 0.96 | 3.19 |
| P7 | 3823 | | 4345 | 2682 | 3179 | 0.96 | 3.61 |
| P7 | 3660 | | 4284 | 2657 | 3174 | 0.98 | 1.99 |
| P7 | 3759 | | 4304 | 2673 | 3152 | 0.97 | 2.68 |
| **P7** | **3769** | | **4318** | **2657** | **3128** | **0.97** | **2.51** |
| P7 | 3712 | | 4242 | 2656 | 3125 | 0.97 | 2.68 |
| P7 | 3806 | | 4344 | 2665 | 3144 | 0.97 | 2.95 |
| P7 | 3747 | | 4286 | 2653 | 3123 | 0.97 | 2.62 |
| P7 | 3692 | | 4152 | 2667 | 3125 | 0.96 | 3.55 |
| P7 | 3597 | | 4066 | 2653 | 3107 | 0.97 | 3.13 |
| P7 | 3588 | | 4047 | 2665 | 3102 | 0.97 | 2.85 |
| P7 | 3725 | | 4172 | 2682 | 3167 | 0.95 | 4.40 |
| P7 | 3684 | | 4175 | 2668 | 3141 | 0.96 | 3.31 |
| P7 | 3678 | | 4181 | 2670 | 3147 | 0.96 | 3.18 |
| P7 | 3648 | | 4114 | 2645 | 3099 | 0.96 | 3.33 |
| P7 | 3688 | | 4193 | 2662 | 3127 | 0.97 | 2.94 |
| P7 | 3801 | | 4351 | 2658 | 3135 | 0.97 | 2.72 |
| P7 | 3775 | | 4440 | 2645 | 3137 | 0.99 | 1.10 |
